# Age-Dependent Relevance of Endogenous 5-Lipoxygenase Derivatives in Anxiety-Like Behavior in Mice

**DOI:** 10.1371/journal.pone.0085009

**Published:** 2014-01-08

**Authors:** Luciana M. Leo, Suellen Almeida-Corrêa, Claudio A. Canetti, Olavo B. Amaral, Fernando A. Bozza, Fabricio A. Pamplona

**Affiliations:** 1 D’Or Institute for Research and Education, Rio de Janeiro, Brazil; 2 Lab. Neurobiology of Memory, Instituto de Bioquímica Médica, Universidade Federal do Rio de Janeiro (UFRJ), Rio de Janeiro, Brazil; 3 National Center for Bioimaging (CENABIO), Universidade Federal do Rio de Janeiro (UFRJ), Rio de Janeiro, Brazil; 4 Lab. Inflammation, Instituto de Biofísica Carlos Chagas Filho, Universidade Federal do Rio de Janeiro (UFRJ), Rio de Janeiro, Brazil; 5 Lab. Immunopharmacology, Instituto Oswaldo Cruz, Fundação Oswaldo Cruz (FIOCRUZ), Rio de Janeiro, Brazil; Max Planck Institute of Psychiatry, Germany

## Abstract

When 5-lipoxygenase (5-LO) is inhibited, roughly half of the CNS effect of the prototypic endocannabinoid anandamide (AEA) is lost. Therefore, we decided to investigate whether inhibiting this enzyme would influence physiological functions classically described as being under control of the endocannabinoid system. Although 5-LO inhibition by MK-886 reduced lipoxin A_4_ levels in the brain, no effect was found in the elevated plus maze (EPM), even at the highest possible doses, via i.p. (10 mg/kg,) or i.c.v. (500 pmol/2 µl) routes. Accordingly, no alterations in anxiety-like behavior in the EPM test were observed in 5-LO KO mice. Interestingly, aged mice, which show reduced circulating lipoxin A_4_ levels, were sensitive to MK-886, displaying an anxiogenic-like state in response to treatment. Moreover, exogenous lipoxin A_4_ induced an anxiolytic-like profile in the EPM test. Our findings are in line with other reports showing no difference between FLAP KO or 5-LO KO and their control strains in adult mice, but increased anxiety-like behavior in aged mice. We also show for the first time that lipoxin A_4_ affects mouse behavior. In conclusion, we propose an age-dependent relevancy of endogenous 5-LO derivatives in the modulation of anxiety-like behavior, in addition to a potential for exogenous lipoxin A_4_ in producing an anxiolytic-like state.

## Introduction

Our laboratory recently described that the 5-lipoxygenase (5-LO) derivative lipoxin A_4_ is a positive allosteric modulator of CB1 cannabinoid receptors [1]. Such mechanism had not been previously described for this or other endogenous lipids, and occurs in addition to the well-described interaction of lipoxin A_4_ with ALX receptors [2]. Interestingly, when 5-LO is inhibited, roughly half of the CNS effect of the prototypic endocannabinoid anandamide (AEA) is lost [1]. Therefore, we decided to investigate whether 5-LO inhibition would influence physiological functions classically described as being under control of the endocannabinoid system (for a recent review, see [3]). This research line was started with anxiety, which is deeply influenced by the endocannabinoid system, and particularly by AEA. We hypothesized that if 5-LO inhibition reduces AEA effects in the brain, mice treated with 5-LO inhibitors would show increased anxiety-like behavior, similarly to what occurs with mice treated with CB1 receptor antagonists [4] and contrary to what occurs after enhancement of AEA levels by URB597 [5]. However, we could not observe this behavioral phenotype and two recent studies investigating 5-LO knockout mice suggest that lack of effect may indeed be the expected result [6], [7]. On the other hand, exogenous lipoxin A_4_ induced an anxiolytic-like profile in the elevated plus maze (EPM) test.

## Methods

### Animals

Swiss albino and C57BL/6 adult (3 months) and aged (12 months) male mice have been used in this study. 5-LO knockout female mice were provided by Fundação Oswaldo Cruz and kept in the animal facilities of the National Center for Bioimaging (CENABIO) at Universidade Federal do Rio de Janeiro. The animals were maintained under a 12∶12 h light cycle with standard rodent chow and filtered water provided *ad libitum*. All procedures were carried out in strict accordance with international recommendations for the Care and Use of Laboratory Animals. The Committee on the Ethics of Animal Experiments of the Universidade Federal do Rio de Janeiro approved our experimental protocols (IBQM058).

### Drugs and Treatment Schedule

MK-886 (Cayman), an inhibitor of the 5-LO activating protein (FLAP) was injected systemically (3–10 mg/kg, i.p.) and intracerebroventricularly (250–500 pmol/2 µl, i.c.v.). This drug had already been tested in our previous paper, and a dose of 3 mg/kg was sufficient to inhibit half of AEA-induced catalepsy [1]. Doses ranging from 3 to 10 mg/kg also influence mouse behavior in the forced swim test [8]. The direct 5-LO inhibitor zileuton (Cayman) was given orally in a selected high dose (40 mg/kg, p.o.), which is roughly 25% above the dose known to inhibit 50% of the enzyme activity (ED_50_) in the periphery [9]. Both drugs were injected 1 h before behavioral experiments and 10% DMSO was used as vehicle. AM251 (1–3 mg/kg) and lipoxin A_4_ (1–10 µg) were injected i.p. 30 min before testing.

### Behavioral Test

The elevated plus maze (EPM) test evaluates anxiety-like behavior in rodents, and was conducted with the same protocol previously shown by our group [10] and others [4] to be sensitive to manipulations of the endocannabinoid system. A positive control with the CB1 antagonist AM251 was also performed to confirm that the experimental settings are sensitive to manipulations inducing an anxiogenic-like state. In brief, each mouse was placed in the center of a plus-shaped elevated maze, which has two open arms and two enclosed arms, and is allowed to explore the apparatus for 5 min. The time spent in the open arms and the number of entries in these arms is regarded as an inverse measure of anxiety, whereas the number of closed arm entries is regarded as a measure of total locomotion [11]. The experiments have been conducted under approximately 300–400 Lux illumination.

### Immunodetection of Lipoxin A_4_ by ELISA

Blood plasma has been collected from Swiss and 5-LOX KO mice and applied into a double-sandwich ELISA kit (Oxford Biomedical Research), read at 650 nm, and expressed as (ng/ml of plasma). To evaluate if 5-LO inhibition would prevent the increase of lipoxin A_4_ levels in a condition that induces on demand endocannabinoid synthesis, we injected Swiss mice with MK-886 (10 mg/kg, i.p.) 30 min before kainic acid (30 mg/kg, i.p.) to measure lipoxin A_4_ in the hippocampus. Brains were harvested and the hippocampus has been dissected 30 min after kainic acid administration. The ELISA procedure followed the supplier’s instruction and has been previously described, and lipoxin A_4_ levels were expressed as ng/mg of wet tissue weight [1].

### Statistics

The results were compared by one-way ANOVA using treatment as independent variable for the multiple doses schedules. Significant differences have been compared by the Newman-Keuls post-hoc test. Two-group comparisons have been performed by Student’s T-test. Statistical significance was set as p<0.05.

## Results

**Figure 1:**
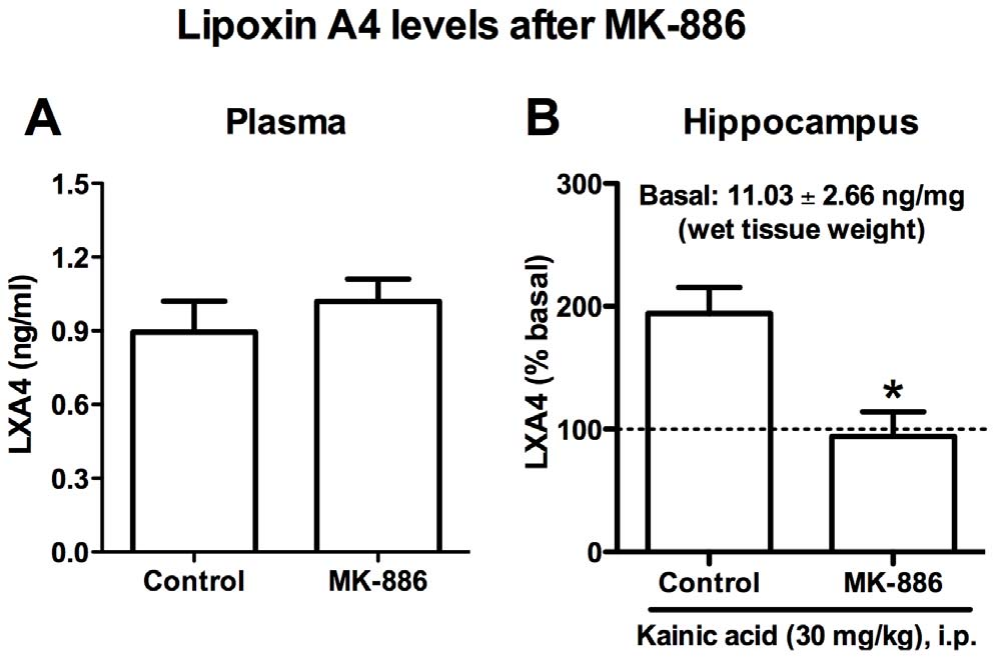
MK-886 prevents on demand synthesis of lipoxin A_4_ in adult Swiss mice. Treatment with (MK-886 10 mg/kg, i.p.) did not reduce (A) basal circulating levels of lipoxin A_4_ (n = 8−12). However, (B) MK-886 (10 mg/kg, i.p.) successfully prevented the increase in lipoxin A_4_ levels induced by kainic acid in the brain of adult Swiss mice (n = 6°−7). Data are presented as mean ± SEM and were analyzed by two-tailed Student’s t test. In “B” data was normalized by basal levels found in control animals. *p<0.05 vs control. LXA_4_: Lipoxin A_4_.

Our first approach was to inject MK-886, which is a 5-LO activating protein (FLAP) inhibitor, generating an indirect inhibition of 5-LO. This drug had already been tested in our previous paper, and a dose of 3 mg/kg was sufficient to inhibit half of AEA-induced catalepsy [1]. Doses ranging from 3 to 10 mg/kg also influence mouse behavior in the forced swim test [8]. Therefore, we tried to observe whether pharmacological 5-LO inhibition in fact reduces circulating lipoxin A_4_ levels (Fig. 1). In basal circumstances, one hour after MK-886 (10 mg/kg, i.p.) injection, there was no evidence of reduced plasmatic levels of lipoxin A_4_ in the plasma of adult male Swiss mice [t(18) = 0.73, p = 0.47]. On the other hand, when production of lipoxin A_4_ was induced by kainic acid administration [p<0.05 vs control] a significant prevention of induction by MK-886 was observed in the hippocampus [t(11) = 3.44, p = 0.006].

To evaluate the impact of 5-LO inhibition on anxiety-like behavior, we injected MK-886 (3–10 mg/kg, i.p.) in young adult male Swiss mice and 1 h later they were tested in the EPM test. There was no effect of MK-886 on open arm time (p = 0.90), open arm entries (p = 0.25), closed arm time (p = 0.85), or closed arm entries (p = 0.34) (Table 1).

**Table 1:** Treatment with MK-886 does not influence anxiety-like behavior in the EPM test.

Mouse strain (n)	Treatment/Statistics	Open Arm Time	Closed Arm Time	Open Arm Entries	Closed Arm Entries
Swiss (10)	Vehicle i.p.	8.4±3.3	138.4±7.3	1.6±0.7	13.8±1.1
	MK-886 3 mg/Kg i.p.	5.5±2.3	150.3±7.7	1.2±0.4	15.0±1.0
	MK-886 10 mg/Kg i.p.	7.6±2.6	160.2±6.2	1.2±0.4	13.6±0.9
	ANOVA	F(27,2) = 0.29, p = 0.75	F(27,2) = 2.3, p = 0.11	F(27,2) = 0.22, p = 0.80	F(27,2) = 0.58, p = 0.57
C57BL/6 (8–9)	Vehicle i.p.	54.0±14.5	157.1±25.3	6.5±1.9	8.1±1.7
	MK-886 3 mg/Kg i.p.	44.4±18.7	166.8±17.7	3.0±1.0	6.2±0.7
	MK-886 10 mg/Kg i.p.	53.4±16.9	152.1±14.0	4.8±1.3	8.0±0.4
	ANOVA	F(23,2) = 0.10, p = 0.90	F(23,2) = 0.16, p = 0.85	F(23,2) = 1.48, p = 0.25	F(23,2) = 1.13, p = 0.34
Swiss (9–10)	Vehicle i.c.v.	20.2±7.2	146.8±11.8	3.1±1.1	12.1±0.5
	MK-886 250 pmol/2 µl i.c.v.	38.8±12.1	134.7±11.4	5.1±1.5	13.4±1.2
	MK-886 500 pmol/2 µl i.c.v.	32.9±9.0	123.0±11.9	4.7±1.0	13.9±0.8
	ANOVA	F(26,2) = 0.99, p = 0.38	F(26,2) = 0.35, p = 0.36	F(26,2) = 0.81, p = 0.45	F(26,2) = 1.29, p = 0.29
Swiss (6–8)	Vehicle i.p.	15.6±3.8	147.0±7.4	3.0±0.7	14.7±1.6
	AM251 1 mg/Kg i.p.	5.5±3.0*	141.8±11.8	0.7±0.2**	10.5±2.5
	AM251 3 mg/Kg i.p.	3.6±1.7*	202.9±14.5**	0.5±0.2**	8.9±1.9
	ANOVA	F(2,18) = 5.2, p = 0.02	F(2,18) = 8.3, p = 0.003	F(2,18) = 10.65, p = 0.009	F(2,18) = 2.4, p = 0.12

Mice were treated with MK-886 via i.p. or i.c.v. route and tested on the EPM. There was no significant difference in anxiety-like behavior among groups in either mouse strain (Swiss or C57BL/6) or administration route (i.p. or i.c.v.). AM251, a CB1 antagonist, was used as positive control. Data are presented as Mean ± SEM. Statistical analysis was carried out by ANOVA followed by Duncan’s post hoc test.

p<0.05 vs vehicle.

p<0.01 vs vehicle.

As information about MK-886 bioavailability in the CNS is scarce [12] – even though the drug has been shown to act systemically – we also injected MK-886 via i.c.v. route, in order to overcome any putative obstacle in drug absorption. MK-886 was injected at the maximal possible doses (250–500 pmol/2 µl, i.c.v.) considering its solubility in DMSO 10% (used as vehicle). However, once again, there was no effect of MK-886 in the EPM test (Open arm time: p = 0.38; Open arm entries: p = 0.46; Closed arm time: p = 0.35; Closed arm entries: p = 0.29) (Table 1). MK-886 was also ineffective in C57BL/6 mice (p>0.05 in all parameters) (Table 1). A similar approach was tested in the dark-light box, without any effect of 5-LO inhibitors (MK-886 and zileuton) (Table S1).

Mouse behavior in the current EPM setting was sensitive to the CB1 cannabinoid antagonist AM251, used as a positive control in the current study. CB1 blockade affected both the time spent [F(18,2) = 5.20; p = 0.02] and entries [F(18,2) = 10.65; p = 0.002] in open arms, as well as time spent in closed arms [F(18,2) = 8.32; p = 0.003]. Both 1 mg/kg and 3 mg/kg of AM251 caused anxiogenic-like behavior in the EPM (p<0.05), while 3 mg/kg apparently also reduced locomotion (Table 1).

**Figure 2:**
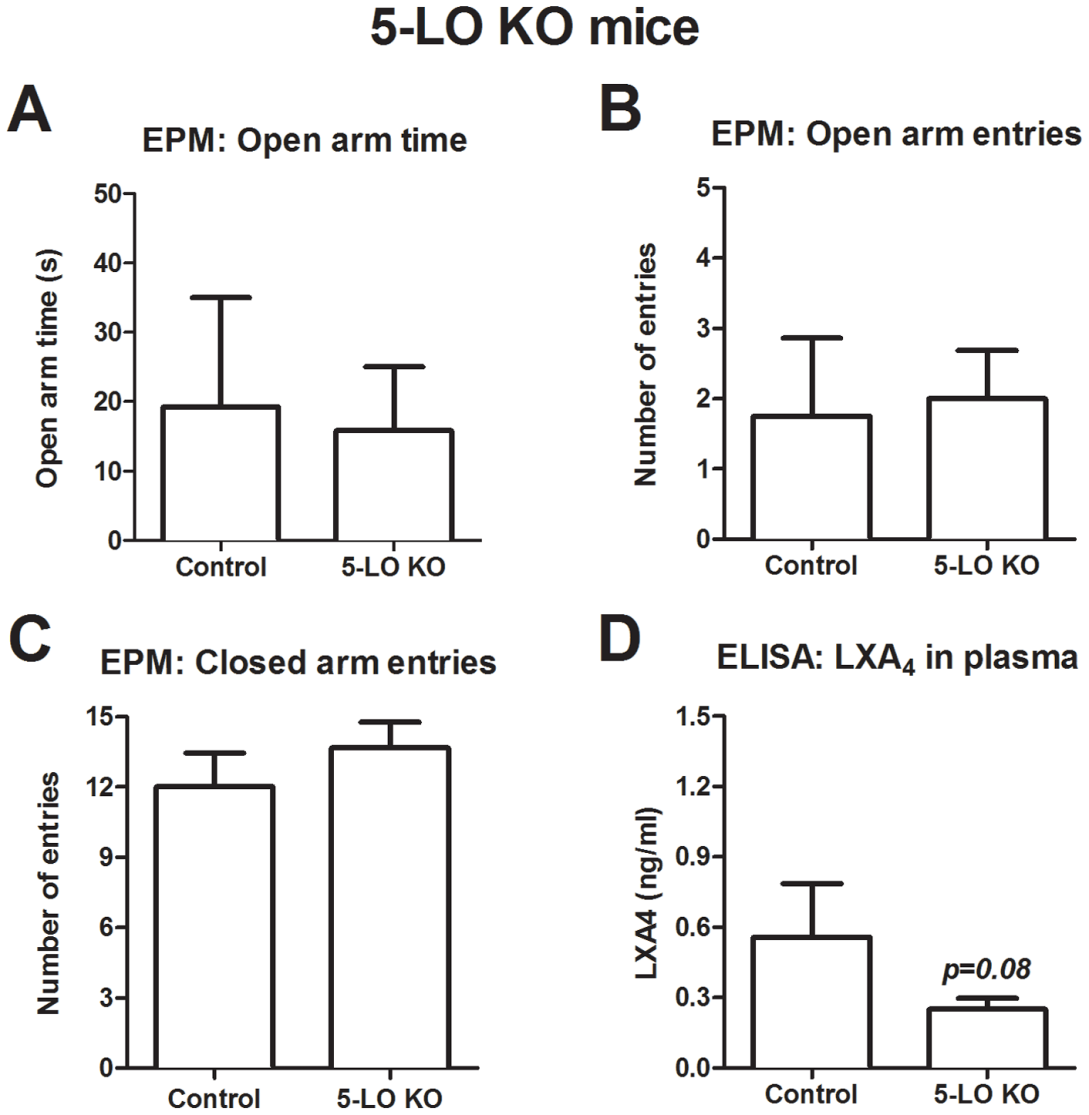
5-LO knockout mice exhibit unaltered anxiety-like behavior in the EPM test. No difference was observed between control and 5-LO knockout mice as to (A) open arm time, (B) open arm entries or (C) closed arm entries (n = 8−12). (D) 5-LO knockout mice show significantly reduced lipoxin A_4_ in plasma (n = 8−10). Data are expressed as mean ± SEM and statistical analysis was performed by two-tailed or one-tailed Student’s t test, respectively. KO: knockout, 5-LO: 5-lipoxygenase enzyme.

Similarly to what occurred with pharmacological 5-LO inhibition, genetic deletion of this enzyme did not induce significant behavioral alteration in the EPM test, as shown previously by other groups in young adult mice [6], [7], [8], [13]. Adult female 5-LO KO mice presented a behavioral profile similar to wild-type controls, at least as far as time spent [t(18) = 0.20, p = 0.84] and entries [t(18) = 0.20, p = 0.84] in open arms and time spent [t(18) = 1.27, p = 0.22] and entries [t(18) = 0.93, p = 0.36] in closed arms are regarded. Similarly, 5-LO KO mice presented a tendency towards reduced plasmatic lipoxin A_4_ levels compared to controls [t(16) = 1.48, p = 0.08] (Fig. 2). As already reported in the literature, basal lipoxin A_4_ levels are slightly reduced in the 5-LO KO compared to control animals, but the difference becomes quite evident when lipoxin A_4_ synthesis is artificially induced, for instance, by a pathogen infection [14].

**Figure 3:**
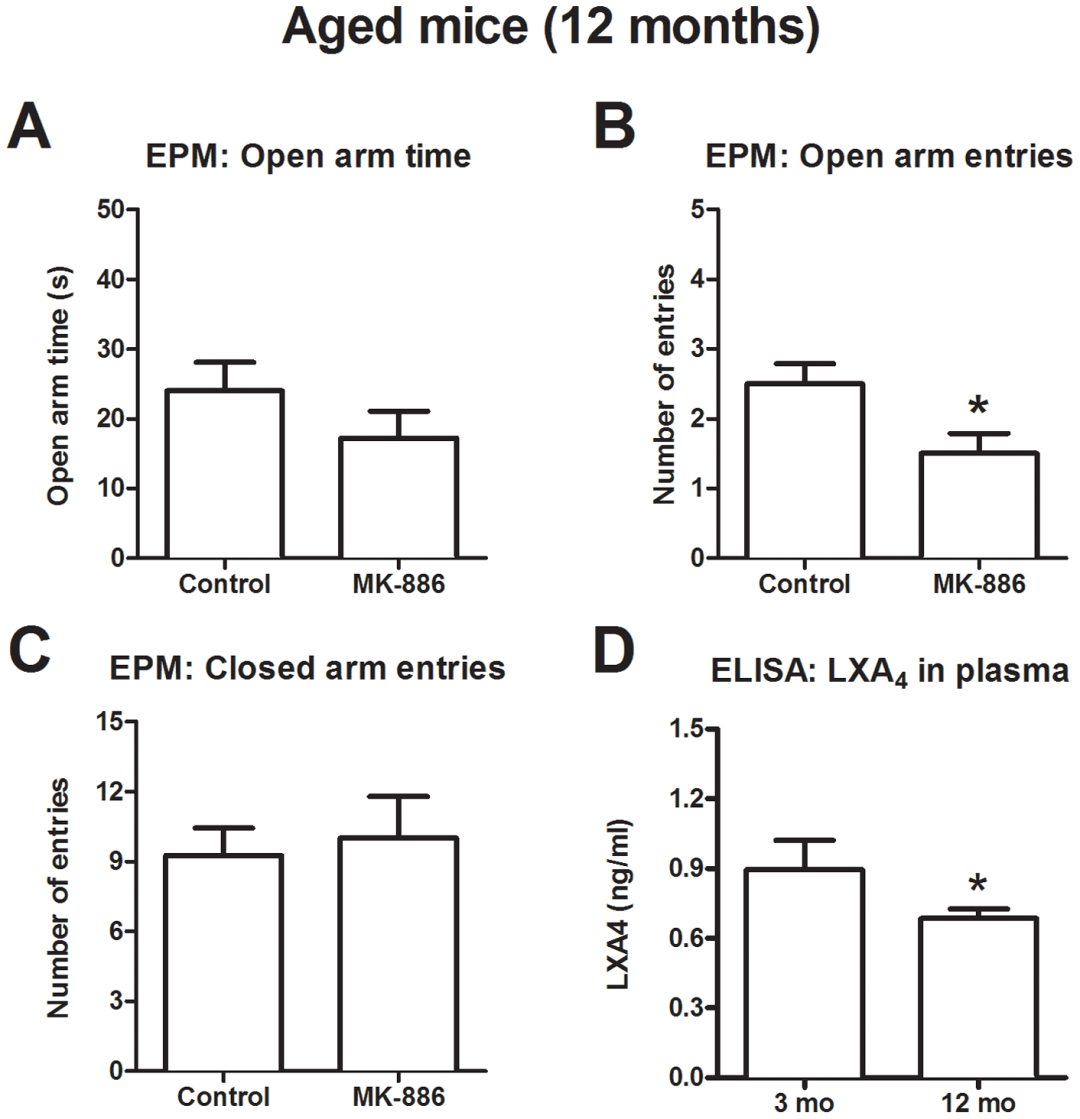
MK-886 induced anxiogenic-like behavior in 12-month-old Swiss mice in the EPM test. After treatment with MK-886 (10 mg/kg, i.p.) aged mice displayed a tendency toward reduced (A) open arm time and a significant decrease in the number of (B) entries in open arms, while no changes were observed in (C) entries in closed arms (n = 4). (D) Twelve-month-old mice also show reduced lipoxin A_4_ levels in plasma compared to 3-month-old mice (n = 12−15). Data are presented as mean ± SEM, statistical analysis was carried out by two-tailed Student’s t test. *p<0.05 vs vehicle. mo: months.

Curiously, the absence of 5-LO seems to influence anxiety-like behavior at a later time point during the aging process [14]. Therefore, to confirm this data using a pharmacological approach, we treated 12-month-old male Swiss mice with the same dose of MK-886 used in the previous experiments (10 mg/kg, i.p.). A very small number of aged animals were available for the experiment (n = 4) but, to our surprise, a single administration of this 5-LO inhibitor induced a slight anxiogenic-like effect in the EPM. Open arm entries were reduced [t(6) = 2.45, p = 0.05] and time spent in open arms were not significantly different [t(6) = 1.2, p = 0.27] (Fig. 3). Importantly, the same behavioral profile was consistent in the light-dark box, as shown in the supplement (Table S1) (reduced time in bright side [t(7) = 3.32, p = 0.01]; increased time in the dark side [t(7) = 3.11, p = 0.02] and no changes in transitions [t(7) = 0.57, p = 0.59]). Moreover, 12-month old mice showed reduced lipoxin A_4_ levels compared to adult controls [t(25) = 1.72, p = 0.05].

**Figure 4:**
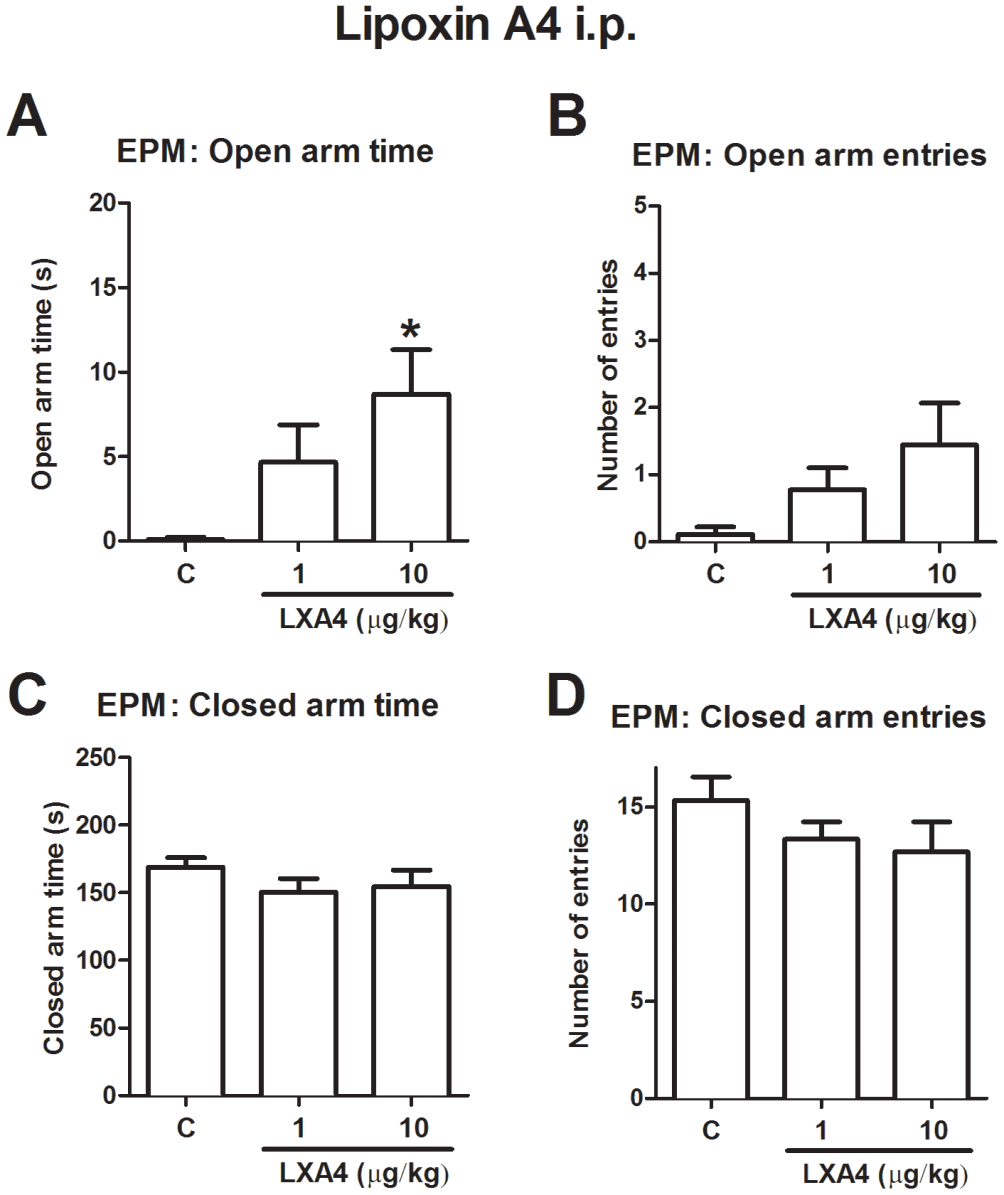
Anxiolytic-like effect of exogenous Lipoxin A_4_ in the EPM test. After a single dose of Lipoxin A_4_ (10 ug/kg, i.p.), adult Swiss mice showed a significant increase in (A) open arm time. No difference was observed in (B) entries in open arms, (C) time in closed arms or (D) entries in closed arms. Data are presented as mean ± SEM (n = 9). Statistical Analysis was performed by ANOVA followed by Newman-Keuls post hoc test. *p<0.05 vs vehicle.

Although the previous results suggest that endogenous 5-LO derivatives’ impact on emotionality is age-dependent, we wanted to know whether exogenous systemic application of lipoxin A_4_ would induce an anxiolytic-like effect in the EPM. That is what would be expected given its recently described enhancement of AEA potency due to positive allosteric modulation of CB1 receptors [1]. Indeed, lipoxin A_4_ (1–10 µg/kg, i.p.) increased time spent [F(24,2) = 4.61; p = 0.02] and a trend towards entries [F(24,2) = 2.62; p = 0.09] in open arms, without affecting time spent [F(24,2) = 0.96; p = 0.39] and entries (F(24,2) = 1.25; p = 0.31) in closed arms in the EPM test (Fig. 4).

## Discussion

The current set of experiments presents evidence that endogenous 5-LO derivatives’ influence on anxiety-like behavior in mice is age-dependent, although exogenous lipoxin A_4_ induces anxiolytic-like effects. This is important under the light of a recent report showing that lipoxin A_4_ is an endogenous positive allosteric modulator of CB1 cannabinoid receptors, which enhances the potency of the endocannabinoid AEA [1]. Since a potential therapeutic exploitation of AEA signaling has already been proposed by many authors [4], [15], [16], this paper suggests that positive allosteric modulation of CB1 receptors is a valid mechanism to achieve this goal, although this modulation might not be endogenously relevant in physiological circumstances.

In the current study, either pharmacological inhibition or genetic deletion of 5-LO did not influence anxiety-like behavior in the EPM in adult animals, although 5-LO inhibition induced an anxiogenic-like state in aged mice. The 5-LO inhibitor could prevent induction of lipoxin A_4_ levels in the brain, but did not change basal plasmatic levels. As kainic acid induces on demand endocannabinoid synthesis in the hippocampus [17], [18], these results suggest that lipoxin A_4_ may also be released in a condition in which increased endocannabinoid levels are observed. Likewise, 5-LO KO presented a tendency towards reduced basal lipoxin A_4_ levels, which is consistent with the current literature [14]. Aged animals displayed reduced lipoxin A_4_ in the plasma and anxiogenic-like behaviors in the EPM, showing that this molecule may influence emotionality under age-related conditions.

Two recent papers have studied the effect of genetic deletion of 5-LO on anxiety using the EPM [6], [7]. In the first paper, the authors investigated whether female 5-LO KO (commercially available at Jackson Laboratory) expressed behavioral alterations in this task compared to control mice. One interesting point of this article is that they tested mice of 3 to 12 months of age. Our results are in line with their initial observations that 3-month-old KO mice exhibited a behavioral profile very similar to that of control mice in the EPM [6]. However, when older female mice were tested, different results were observed. Both 6 or 9-month-old female mice displayed an anxiogenic-like profile compared to control mice, in line with our initial expectations on the outcome of 5-LO inhibition [6]. In the second paper, the authors investigated the same issue using FLAP knockout mice from Jackson Laboratory [7]. Similar to what was observed in 5-LO KO, FLAP KO mice displayed “normal” behavior in the EPM at the age of 3 months, but displayed increased anxiety-like behavior with age (statistically significant at 12 months of age). This behavior alteration was accompanied by reduced locomotion in the EPM, but not by alterations in spontaneous alternation task in the Y-maze [7]. Remarkably, Uz et al have reported results in an opposite direction: at the age of 2–3 months, male 5-LO KO mice showed *reduced* anxiety in the EPM and marble burying tests, without alterations in locomotor activity or spontaneous alternation in the T-maze [13]. The difference between these studies certainly deserves careful examination. While the KOs used by Joshi and Pratico were generated on a C57BL/6 background (and therefore C57BL/6 mice were used as controls), Uz et al used KO mice generated on a hybrid background, an F2 generation of B6/129 mice as controls. In the former, there was no difference between C57BL/6 mice and KOs in the EPM, while an unexpected anxiolytic effect was reported in the latter. However, in the same paper, Uz et al showed that their control mice displayed behavioral differences when compared to C57BL/6 and 129P3 parental strains [13]. This effect supports the notion that the interpretation of results is potentially flawed by a floor effect, since the B6129SF2 mice shows a marked anxiogenic-like profile. The strain background issue is one of the reasons why we prefer to use outbred mouse strains to perform pharmacological research. We believe that any drug potentially intended for translational research must at least induce an effect higher than the “natural” variance observed in a heterogeneous population. Of course, it is not possible, nor acceptable, to use outbred mice as controls for knockout strains.

All in all, our negative findings on 5-LO modulation and anxiety-like behaviors are in line with most - if not all - data available on young adult mice carrying genetic ablation of 5-LO-related genes. An overall analysis of these data may suggest that 5-LO derivatives are important for anxiety modulation only under specific circumstances, such as gender (e.g. only important in females), during specific phases of development (e.g. aging) or pathological states. That would explain why we could not observe any effect of pharmacological 5-LO inhibition using healthy adult male mice, but succeeded to do so using 12 month-old Swiss mice. This is a relevant finding, as it was not previously clear whether the anxiogenic-like effect observed in aged 5-LO KO mice was due to the age in which the behavioral tests were performed or to the cumulative effects of the gene knockout over a long period of time. One limitation of our study is that drug treatment was not chronic/repeated, which might yield a more potent 5-LO inhibition and then influence behavior. However, data from the 5-LO KO mice further support data from our pharmacological approach.

Our results suggest that this may be related to a time point in the aging process where lipoxin A_4_ is naturally reduced. There is evidence of a similar age-related reduction in lipoxin A_4_ levels in humans (Gangemi et al, 2005). In this sense, it would be interesting to explore the potential application of lipoxin A_4_ (or derived synthetic molecules) as replacement therapy in aged individuals. This paper shows that, at least as far as anxiety-like behaviors are regarded; using exogenous lipoxin stable analogs might be a useful strategy to reduce anxiety-like behaviors.

## Supporting Information

Table S1 **In the Light-Dark Box task, MK-886 treatment induces anxiogenic-like behavior in aged mice, but not in 3-month-old nor in 5-LO knockout mice.** After treatment with 5-LO inhibitors (MK-886 or Zileuton), adult (3 months) or aged (12 months) mice were tested in the Light-Dark box task. Three-month-old mice did not show altered anxiety-like behavior in response to either treatment, whereas 12-month-old mice displayed an anxiogenic-like behavior, as measured by reduced time spent in bright side and increased time spent in dark side. Adult 5-LO knockout mice (5-LO KO) did not show any difference in anxiety-like behavior compared to control mice. Data are presented as Mean ± SEM. *p<0.05 vs vehicle.
(DOCX)
