# Supplementary material for: Age-Dependent Relevance of Endogenous 5-Lipoxygenase Derivatives in Anxiety-Like Behavior in Mice
Source: PLoS One. 2014 Jan 8;9(1):e85009. doi: 10.1371/journal.pone.0085009 (PMC3885659; doi:10.1371/journal.pone.0085009)
Supplement: Table S1 — In the Light-Dark Box task, MK-886 treatment induces anxiogenic-like behavior in aged mice, but not in 3-month-old nor in 5-LO knockout mice. After treatment with 5-LO inhibitors (MK-886 or Zileuton), adult (3 months) or aged (12 months) mice were tested in the Light-Dark box task. Three-month-old mice did not show altered anxiety-like behavior in response to either treatment, whereas 12-month-old mice displayed an anxiogenic-like behavior, as measured by reduced time spent in bright side and increased time spent in dark side. Adult 5-LO knockout mice (5-LO KO) did not show any difference in anxiety-like behavior compared to control mice. Data are presented as Mean ± SEM. *p<0.05 vs vehicle. (DOCX) [file pone.0085009.s001.docx]

Table S1. In the Light-Dark Box task, MK-886 treatment induces anxiogenic-like behavior in aged mice, but not in 3-month-old nor in 5-LO knockout mice.

| **Mouse strain, age (n)** | **Treatment/Statistics** | **Time in bright side** | **Time in dark side** | **Transitions** |
| --- | --- | --- | --- | --- |
| Swiss, 3 months (8-9) | Vehicle i.p. | 20.6 ± 2.8 | 62.9 ± 3.5 | 11.4 ± 1.9 |
|  | MK-886 3mg/Kg i.p. | 30.1 ± 3.5 | 54.2 ± 4.3 | 16.1 ± 1.8 |
|  | MK-886 10mg/Kg i.p. | 23.5 ± 2.7 | 60.9 ± 2.2 | 16.7 ± 2.2 |
|  | ANOVA | F(2,23)=0.19, p=0.82 | F(2,21)=1.67, p=0.21 | F(2,21)=0.48, p=0.63 |
| Swiss, 3 months (7-9) | Vehicle i.p. | 113.0 ± 8.1 | 150.6 ± 10.6 | 13.4 ± 1.4 |
|  | MK-886 250pmol/2µl i.c.v. | 109.8 ± 10.1 | 155.0 ± 11.1 | 14.4 ± 2.6 |
|  | MK-886 500pmol/2µl i.c.v. | 126.7 ± 8.7 | 128.9 ± 10.4 | 11.8 ± 1.8 |
|  | ANOVA | F(2,21)=1.00, p=0.38 | F(2,21)=1.67, p=0.21 | F(2,21)=0.48, p=0.63 |
| Swiss, 3 months (9-10) | Vehicle p.o. | 142.8 ± 6.8 | 157.2 ± 6.8 | 14.0 ± 1.1 |
|  | Zileuton 40mg/Kg p.o. | 126.0 ± 19.7 | 174.0 ± 19.7 | 12.4 ± 1.8 |
|  | Two-tailed Student’s t test | t(17)=0.83, p=0.41 | t(17)=0.83, p=0.41 | t(17)=0.75, p=0.46 |
| Swiss, 12 months (4-5) | Vehicle i.p. | 61.5 ± 4.6 | 21.6 ± 4.4 | 6.4 ± 2.6 |
|  | MK-886 10mg/Kg i.p. | 34.1 ± 7.3* | 48.8 ± 8.1* | 15.8 ± 4.0 |
|  | Two-tailed Student’s t test | t(7)=3.33, p=0.01 | t(7)=3.11,p=0.02 | t(7)=0.57, p=0.59 |
| 5-LO KO, 3 months (8-12) | Control | 65.6 ± 9.8 | 163.3 ± 11.0 | 14.9 ± 2.3 |
|  | 5-LO KO | 85.7 ± 5.4 | 177.8 ± 7.0 | 16.2 ± 1.4 |
|  | Two-tailed Student’s t test | t(18)=1.94, p=0.07 | t(18)=1.17, p=0.25 | t(18)=0.50, p=0.62 |

After treatment with 5-LO inhibitors (MK-886 or Zileuton), adult (3 months) or aged (12 months) mice were tested in the Light-Dark box task. Three-month-old mice did not show altered anxiety-like behavior in response to either treatment, whereas 12-month-old mice displayed an anxiogenic-like behavior, as measured by reduced time spent in bright side and increased time spent in dark side. Adult 5-LO knockout mice (5-LO KO) did not show any difference in anxiety-like behavior compared to control mice. Data are presented as Mean ± SEM. *p<0.05 vs vehicle.
